# Screening halotolerant bacteria for their potential as plant growth-promoting and coal-solubilizing agents

**DOI:** 10.1038/s41598-025-98005-z

**Published:** 2025-04-16

**Authors:** Nuraly Akimbekov, Ilya Digel, Bekzat Kamenov, Nazym Altynbay, Kuanysh Tastambek, Jian Zha, Atakan Tepecik, Svetlana K. Sakhanova

**Affiliations:** 1 Sustainability of Ecology and Bioresources, Al-Farabi Kazakh National University, Al-Farabi Ave. 71, 050040 Almaty, Kazakhstan; 2 Scientific-Practical Center, West Kazakhstan Marat Ospanov Medical University, Maresyev Str. 68, 030019 Aktobe, Kazakhstan; 3 Ecology Research Institute, Khoja Akhmet Yassawi International Kazakh-Turkish University, Sattarhanov Str. 29, 161200 Turkistan, Kazakhstan; 4 Institute for Bioengineering, Aachen University of Applied Sciences, Heinrich-Mussmann-Straße 1, 52428 Jülich, Germany; 5 School of Food and Biological Engineering, Shaanxi University of Science and Technology, Shaanxi, 710021 Xi’an China

**Keywords:** Saline stress, Low-rank coal, Halotolerant bacteria, Coal solubilizing bacteria, Plant growth promotion, Agroecology, Soil microbiology, Plant ecology

## Abstract

The bioconversion of salinized land into healthy agricultural systems by utilizing low-rank coal (LRC) is a strategic approach for sustainable agricultural development. The aims of this study were: (1) to isolate bacterial strains associated with the rhizosphere of native plants in coal-containing soils, (2) to characterize their plant growth-promoting (PGP) and coal-solubilizing capabilities under laboratory conditions and (3) to evaluate their influence on the germination and growth of chia seeds under saline stress. Fourteen bacterial cultures were isolated from the rhizosphere of *Artemisia annua* L. using culture media containing salt and coal. Based on their PGP activities (nitrogen fixation, phosphate solubilization, siderophore and indole-3-acetic acid production), five strains were selected, belonging to the genera *Bacillus*, *Phyllobacterium*, *Arthrobacter*, and *Pseudomonas*. Solubilization assays were conducted to confirm the ability of these strains to utilize coal efficiently. Finally, the selected strains were inoculated with chia seeds (*Salvia hispanica* L.) to evaluate their ameliorating effect under saline stress conditions in coal-containing media. Inoculation with *A. subterraneus* Y1 resulted in the highest germination and growth metrics of chia seeds. A positive but comparatively weaker response was observed with *P. frederiksbergensis* AMA1 and *B. paramycoides* Lb-1 as inoculants. Coal inoculated with halotolerant bacteria can serve as the foundation for humified organic matter in salt-affected environments. The selected halotolerant bacteria enhance coal biotransformation while exhibiting PGP traits.

## Introduction

Soil salinization is a severe environmental problem on a global scale, defined by the accumulation of water-soluble salts that negatively impact soil health and plant productivity. Improper water management practices, coupled with soil pollution, lead to increased soil salinity, resulting in the expansion of saline-alkali desert areas and the deterioration of irrigated lands^1,2^. Salinized soil contains higher amounts of cations (Na^+^, K^+^, Mg^2+^, and Ca^2+^), anions (HCO_3_^−^, Cl^−^, NO_3_^−^, SO_4_^2-^, CO_3_^2-^), and very high electrical conductivity (EC) (more than 4 dS m^−1^), which affects the physicochemical and biological properties of soil, decreasing its productivity^3,4^. Changing climatic patterns and the overuse of groundwater also promote soil salinization. Furthermore, the use of saline water for irrigation in agricultural fields due to freshwater shortages is another factor that accelerates soil salinization. Currently, approximately 20% of cultivated land and 50% of irrigated land worldwide are affected by salinity^5,6^. By the end of 2050, approximately half of the fertile agricultural land could be salinized, further threatening agricultural production across the world^7^.

The rehabilitation and remediation of salt-affected soils have already been addressed by various technologies, including salt leaching and the application of amendments such as gypsum and sulfuric acid. In saline-sodic soils, gypsum is a commonly used amendment to maintain the physical and hydraulic properties of soil. The combination of gypsum, sulfur, and compost has also been applied; however, the high cost of sulfur remains a significant obstacle. Conventional saline soil reclamation methods such as leaching, scraping and flushing, have limited success and may adversely affect agroecosystems^8,9^.

The incorporation of various sources of humified organic matter (HOM) and humic substance sources into soil may mitigate the detrimental consequences of salt accumulation on salt-sensitive crops^10^. HOM improves soil structure, aggregation, hydraulic conductivity, nutrient retention and cation exchange capacity (CEC)^3^. Soil amendment with organic matter is often the most successful practice due to its low cost and wide availability. Several organic amendments, such as manure, vermicompost, biochar and biosolids, can increase soil organic matter content. In addition, the application of new technologies, including biofertilizers (bacteria, algae and fungi) and biopolymers, stimulates microbial and enzymatic activities in soils, improving the properties of salt-affected ecosystems^4,10^.

Low-rank coals (LRC), such as lignite (brown coal) and leonardite, are rich in a wide range of macro- and microelements and are an excellent source of HOM. LRC is not economically feasible for energy and industrial applications due to their soft and friable consistency, opaque appearance, high ash and moisture content, as well as low fixed carbon and calorific value. However, LRC contains a high concentration of oxygen-containing functional groups, a large proportion of macropores and high dispersion of inorganic catalytic constituents^11^. The oxygen-containing functional groups in LRC enable it to remove cations from solutions through ion exchange. The CEC of LRC primarily arises from deprotonated carboxyl and phenolic hydroxyl functional groups, which readily form complexes with cationic species. Furthermore, due to its chemical composition, LRC can function as a suitable nutrient medium for salt-tolerant microorganisms, promoting their growth while releasing substantial amounts of HOM through various metabolic mechanisms^12,13^. These microorganisms may, in turn, exhibit unique physiological properties associated with plant growth-promoting (PGP) abilities.

The aims of this study were (a) to isolate bacterial strains from the rhizosphere of native plants grown in salt-affected soil from abandoned coal spoil-heaps, (b) to characterize their various plant growth-promoting and coal-solubilizing activities, and (c) to evaluate their effects on the germination and initial growth of chia (*Salvia hispanica* L.) under saline stress. To the best of our knowledge, this is the first study to screen isolated salt-tolerant strains for their potential to exhibit both plant growth-promoting and coal-solubilizing traits.

## Materials and methods

### Soil sample collection and characterization

Soil samples for microbial isolation were collected from an abandoned coal slag heap located in a saline zone, Almaty Province, Kazakhstan (44°21′48.7"N 77°57′58.9"E). In particular, bulk soil samples were collected from the areas surrounding the rhizospheres of *Artemisia annua L* plants using the method described by Blouin and Jacquiod^14^. The samples were stored in clean black polyethylene bags at 4 °C and transported to the laboratory for microbial isolation.

The physicochemical properties of the soil were assessed as described by Wilke^15^. The soil pH and electrical conductivity (EC) were measured at a soil-to-water ratio of 1:2 using the conventional technique outlined by Jackson^16^ using a pH/mV meter S400 (The SevenExcellence™, Switzerland).

### Coal sample collection and characterization

The samples were collected from the Ekibastuz coal basin, Pavlodar Province, Kazakhstan (51°41′08.0"N 75°27′26.0"E) according to the method of Dai et al.^17^ and stored in sealed polyethylene bags at 4 °C to prevent contamination and oxidation. The samples were ground and sieved to a particle size less than 0.2 mm prior to use. The proximate and ultimate analyses of LRC were conducted in accordance with American Society for Testing and Materials (ASTM) standards D3172-74 and 5373. The elemental composition was analyzed using a Vario EL cube elemental analyzer (Elementar Analysensysteme GmbH, Germany). The deviations from 100% were attributed to the oxygen content.

### Isolation of microorganisms

Isolation of microorganisms 5 g of soil sample was suspended in Erlenmeyer flasks containing 45 mL of sterile 1% hexametaphosphate, stirred at 200 rpm for 30 min at room temperature to facilitate microbial detachment, and then decanted for 1 h at room temperature to allow solid aggregates to sediment and separate the supernatant with dispersed bacteria^18,19^. After serial dilutions, 100 μL aliquots of the supernatant were plated onto Petri dishes containing the coal nutrient medium of the following composition: KH_2_PO_4_ (1.2 g L^–1^), Na_2_HPO_4_·12H_2_O (10.8 g L^–1^), MgSO_4_·12H_2_O (0.04 g L^–1^), FeSO_4_ 7H_2_O (0.02 g L^–1^), MnCl_2_·4H_2_O (0.02 g L^–1^), NH_4_Cl (3 g L^–1^), coal (50 g L^–1^), agar (15 g L^–1^), and cycloheximide (0.5 g L^–1^), pH 7.0^20^. Cultures were maintained at 30ºC until growth was observed.

### Screening of isolates for plant growth-promoting (PGP) abilities

Various in vitro PGP characteristics of the bacterial isolates were assessed both with and without NaCl (1 M). First, the nitrogen fixation of the isolates was evaluated. The isolates possessing this capability were subsequently assessed for their phosphate solubilizing activity. After determining the strains that exhibited desired activities under all salinity conditions, their siderophore and indole-3-acetic acid (IAA)-like compounds production was assessed. Experiments were performed in triplicate using a 24-h-grown inoculum.

### Nitrogen fixation capacity

Bacterial isolates were cultured on nitrogen-free Jensen’s agar for 5 days at 30 °C with the addition of 1 M NaCl. Positive activity was indicated by the formation of visible bacterial colonies and a change in the medium’s color^21^.

### Phosphate solubilization capacity

The ability of the isolates to solubilize phosphates was tested on Pikovskaya agar containing 1 M NaCl at 30 °C for 7 days. The formation of clear halos around the bacterial colonies indicated positive results^22^.

### Siderophore production

Bacterial cultures were inoculated on chrome azurol-S (CAS) agar plates with 1 M NaCl and incubated for 5–7 days at 30ºC. Positive siderophore production was evidenced by the formation of orange-yellowish halos around the bacterial colonies. CAS agar was prepared according to the method outlined by Schwyn and Neilands^23^.

### IAA-like compounds production

Bacteria were inoculated in nutrient broth supplemented with a 2.5% (v/v) salt solution of the following composition: NaCl (98 g L^−1^), KCl (8.3 g L^−1^) and MgSO_4_ (3.3 g L^−1^) and cultivated at 30 °C with shaking at 150 rpm for 5 days, with and without the addition of tryptophan (0.5 g L^−1^)^24^. Colorimetry was employed to ascertain the synthesis of IAA-like compounds. 1 mL of reagent R1 (12 g L^−1^ FeCl_3_ in 7.9 M H_2_SO_4_) was added to 1 mL of culture supernatant for color development. The mixture was then incubated in the dark at room temperature for 30 min. The absorbance was determined at 530 nm using a UV/Vis spectrophotometer UV-1800 (Shanghai Mapada Instruments Co., Ltd., China), and the measured values were compared with an IAA calibration curve.

### Screening of isolates for coal-solubilizing abilities

Solubilization tests (in submerged and surface variations) were conducted with and without 1 M NaCl to evaluate the coal-solubilizing/utilizing characteristics of the bacterial isolates. Three sets of experiments were performed using a 24-h grown inoculum.

### The coal surface solubilization test (plate test)

This assay was used to assess the ability of isolates to solubilize/utilize coal on a solidified culture medium^25^. The bacterial lawn was grown on MR nutrient agar plates at 37 °C for 48 h. The composition of the MR medium^26^: KH_2_PO_4_ (6.67 g L^–1^), (NH_4_)_2_HPO_4_ (4 g L^–1^), MgSO_4_·7H_2_O (0.8 g L^–1^), citric acid (0.8 g L^–1^), and 5 mL of trace metal solution (per L of 0.5 M HCl): FeSO_4_·7H_2_O (10 g), CaCl_2_ (2 g, ZnSO_4_·7H_2_O (2.2 g), MnSO_4_·4H_2_O (0.5 g), CuSO_4_·5H_2_O (1 g), (NH_4_)_6_Mo_7_O_24_·4H_2_O (0.1 g), Na_2_B_4_O_7_·10H_2_O (0.02 g), pH 7.0. Afterward, autoclaved (121ºC for 15 min) coal particles (~ 3 mm in diameter) were placed on the bacterial lawn and incubated for 7 days. In the case of positive solubilizing activity test results, the coal samples exhibited brown halos.

### The coal submerged solubilization test

This assay was used to evaluate coal solubilization and product formation in a liquid medium. The 0.5 McFarland standard inoculum was grown in shake flasks containing 200 mL of LB medium (10 g L^–1^tryptone, 5 g L^–1^yeast extract, 10 g L^–1^NaCl, pH 7.0.) at 30 °C / under continuous mild (under g-force equal to 3 g) centrifugation for 2 days. Subsequently, 5% (w/v) of powdered sterile coal was added to the culture, which was further cultured for 14 days under identical conditions. On alternate days, culture aliquots were collected, centrifuged at a relative centrifugal force of 11,200 g for 15 min, and then filtered through 0.22 µm nitrocellulose membrane filters (Millipore). The supernatant was diluted 20-fold, and its absorbance at 450 nm was measured using a UV-2600 spectrophotometer (Shimadzu, Japan)^27^.

### Fourier Transform Infrared Spectroscopy (FTIR)

The IR-spectra of the coal biosolubilized products were obtained using an ALPHA II QuickSnap system (Bruker Optics GmbH, Germany). Scans were conducted within the range 400–4000 cm^−1^ with a resolution of 4 cm^−1^. For each sample, a set of spectra was acquired at a rate of 24 spectra per second and averaged for analysis.

### Identification of bacterial isolates

The isolates were identified via sequence analysis of the PCR-amplified 16S rRNA gene. The bacterial DNA was amplified using the universal bacterial primers 27F (5'-AGA GTT TGA TCC TGG CTC AG-3') and 1492R (5'-GGT TAC CTT GTT ACG ACT T-3'). Agarose gel (2%) electrophoresis was employed to analyze the amplified PCR products, which were subsequently purified and sequenced using an ABI 3730 DNA sequencer (Thermo Fisher Scientific, Waltham, MA, USA). Basic Local Alignment Search Tool (BLAST v.2.14.0) was employed via the National Center for Biotechnology Information (NCBI) server (https://blast.ncbi.nlm.nih.gov/Blast.cgi) to compare the recovered sequences with reference sequences from GenBank at the NCBI.

### Biomass production under saline conditions

The cultures were cultivated in 96-well microplates using nutrient broth enriched with 1 M NaCl. 200 μL of 24-h bacterial culture was added to each well. The plates were then incubated at 30 °C and shaken at 150 rpm for 5 days. The optical density at 600 nm was measured using a microplate reader spectrophotometer Infinite® M Nano (Tecan Group AG, Swiss) at 24-h intervals^19^.

### Salinity stress amelioration assay

Following the initial screening, bacteria exhibiting beneficial growth promotion and coal solubilization activities were selected to evaluate their growth-promoting effects on chia seeds. The half-strength Murashige-Skoog medium (MS/2) supplemented with 2 g L^−1^ sucrose and solidified with 0.8% agar was used for this assay. Five bacterial strains were assessed, resulting in six experimental variants, including the negative control (bacteria-free growth medium). Prior to seed treatment, the bacterial strains were cultivated in a nutrient medium at 30 °C in an orbital shaker-incubator at 200 rpm for 24-h. The OD_600_ of the culture was adjusted to a final value of 0.6 (~ 10^8^–10^9^ CFU/mL) for seed bacterization. The treatments were formulated by incorporating NaCl and coal into MS/2 agar. The control treatment was performed using MS/2 without salt and coal supplementation (Table 1).

**Table 1:** Growth conditions of chia seeds under saline stress and coal amendment.

Treatments	NaCl, mM	Coal, g	EC, dS/m
Salt addition	100	–	7.26–8.72
Salt and coal addition	100	7.5	7.01–8.29
Coal addition	–	7.5	2.89–3.76

A NaCl concentration of 100 mM was selected for salinity stress amelioration. The applicable NaCl concentrations relevant to various studies are highly variable, which complicates the identification of effective/optimal concentrations. However, in many studies devoted to the effects of salt stress in plants NaCl concentrations close to 100 mM were used^19,28–32^. The coal concentrations used in biosolubilization studies vary significantly due to their distinct geographical origins and depositional histories. Our previous study^33^ demonstrated that the selected coal is highly susceptible to microbial attack owing to its organic nature and composition. Due to the rich content of humic substances (up to 58%), the calculated application rate of this coal for the germination and development of chia seedlings was established at 7.5 g per 1 L medium.

Prior to the experiments, the surface of chia seeds underwent vapor-phase sterilization by chlorine gas. The seeds were then placed in Petri dishes and positioned inside a 10-L desiccator. A 250 ml glass beaker with 100 mL of household bleach (5.75% NaClO) and 3 mL of hydrochloric acid (37% HCl) was carefully placed next to the seed container. The chlorine gas was allowed to act for 4–6 h after the desiccator was closed and securely sealed. Subsequently, 10 seeds were plated on each MS/2 plate and was inoculated with 20 µL of bacterial culture. The plates were placed in the dark at 4 °C for 24-h for seed stratification. Then, they were placed vertically within a growth chamber, maintained under a photoperiod of 8-h light and 16-h dark at a constant temperature of 23 °C. After seven days, the germination percentage was recorded, and the total plant length (measured from the root tip to the cotyledons) and the length of the primary root (measured from the base of the hypocotyl to the root tip) were assessed for each plant.

### Biocompatibility test

The biocompatibility of the isolated bacterial strains was evaluated using the drop technique, which involves direct co-cultivation on a solid medium, as described by^34^. A 24-h test culture standardized at 10^8^ cells mL^–1^ was applied to the surface of the LB solid medium using a microbiological loop. The culture drop was left until completely absorbed. Further, a drop of the second culture was applied with an offset of 1–2 mm from the edge of the first drop, ensuring that it flowed onto the drop of the first culture. After absorbing the drops, the dishes were incubated at 30 °C for 5 days. The appearance of a growth inhibition halo between the culture droplets indicates an antagonistic relationship between the bacteria.

### Statistical analysis and image processing

Experimental treatments were organized using a fully randomized design with three or five replicates (depending on the performed test). The open-source image processing software FIJI was employed to conduct data collection and image processing^35^. All measured data were assumed to be normally distributed with homogeneous variance errors. If not otherwise specified, the obtained values are shown as mean ± standard deviation. Statistical significance of the observed differences was determined using one-way ANOVA with Tukey’s post-test. Probability levels of 0.05 or less were considered statistically significant.

## Results

### Physicochemical characteristics of soil samples

The physical and chemical properties of the collected soil samples are presented in Table 2. The coal-contaminated soils had a non-sticky, silty texture.

**Table 2:** Main characteristics of soil samples.

Soil physical properties			Soil chemical properties		
Sand, g kg^-1^	Silt, g kg^-1^	Clay, g kg^-1^	pH (1:2)	EC, dS/m	Organic matter, g kg^-1^
64.8	29.4	5.8	7.8	8.03	2.9

### Technical characteristics of coal samples

A comprehensive analysis of the proximal and ultimate parameters was conducted to assess the technical characteristics of the coal samples, as shown in Table 3. Based on the analyses, the samples were classified as low-rank coal (LRC).

**Table 3:** Results of proximate and ultimate analyses of the coal samples.

Ultimate analysis (db, wt%)				Proximate analysis (ar, wt%)				
Moisture, W	Ash, A	Volatile matter, V	Calorific value, Q (MJ/kg)	C	H	N	S	O^diff^
7.1	38.9	23.2	32	77.1	5.08	2.12	0.9	14.8

### Isolation of bacterial cultures

**Fig. 1:**
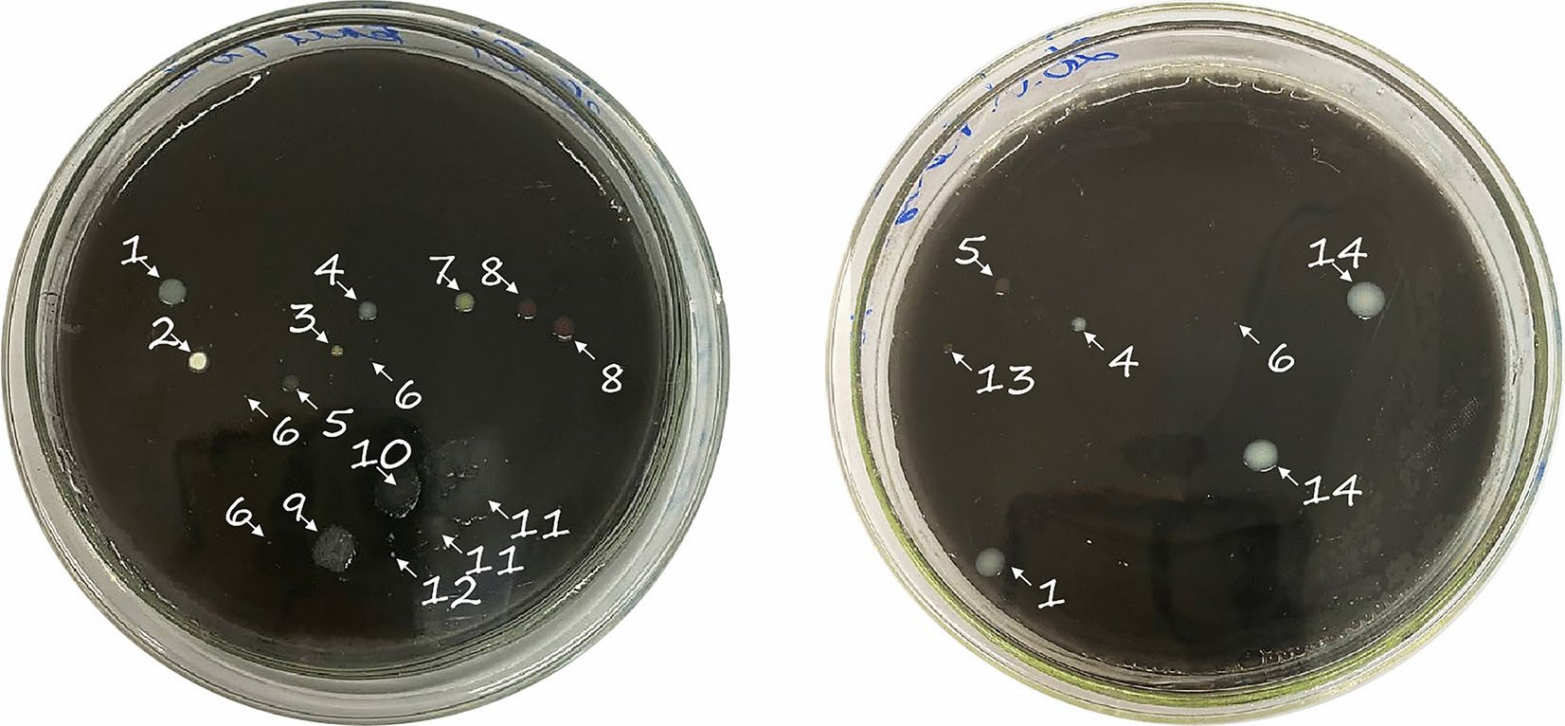
Bacterial colonies grown on coal medium inoculated with soil extract from the rhizospheres of *Artemisia annua* L. The colonies selected for subsequent screening are numerated and marked by arrows.

Fourteen morphologically distinct bacterial colonies were isolated from the coal medium by inoculating soil samples from the rhizosphere of Artemisia annua L. (Fig. 1). Afterward, the isolated bacterial cultures underwent stepwise screening for plant growth-promoting (PGP) properties.

### Testing PGP traits

**Fig. 2:**
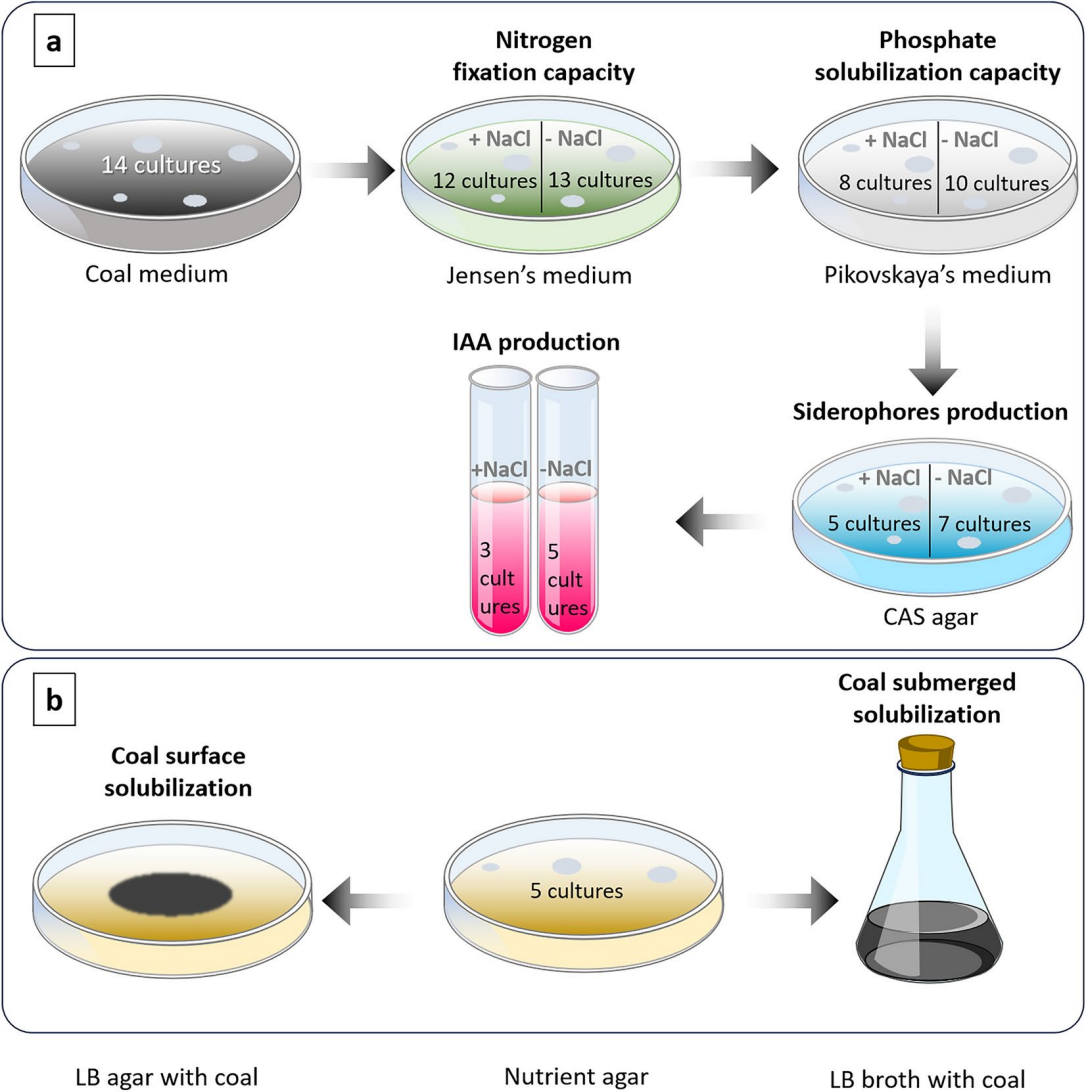
Schematic representation of the stepwise screening process for evaluating plant growth-promoting (PGP) and coal-solubilizing properties of isolated bacterial cultures: (a) Screening for PGP properties; (b) Screening for coal-solubilizing properties. The number of cultures exhibiting each property is indicated.

Of the 14 isolated cultures, 12 were capable of fixing nitrogen in the presence of 1 M NaCl (on the Jensen’s medium) (Fig. 2a). Of these 12 isolates, 10 could solubilize phosphates in NaCl-free Pikovskaya’s medium, while only 8 retained this activity in the presence of NaCl. Among these 8 cultures that could both fix nitrogen and solubilize phosphates, 7 demonstrated siderophore production in CAS medium without NaCl, whereas only 5 were able to produce under high salinity conditions. Ultimately, these siderophore-positive cultures were assessed for their production of IAA-like compounds. All five remaining cultures exhibited the IAA-synthetizing activity in the absence of NaCl, and three of them retained this ability in a saline cultivation medium. Finally, all IAA-like compound-synthetizing cultures were tested for coal-solubilizing activity (Fig. 2b).

#### Strain identification

The five bacterial cultures that displayed all expected PGP activities were identified using 16S rRNA analysis. Sequencing of the corresponding DNA regions revealed that the isolates belonged to four bacterial genera: *Bacillus, Phyllobacterium, Arthrobacter* and *Pseudomonas* (Table 4). The corresponding NCBI links and the phylogenetic trees of the strains are provided in the supplementary files section (GenBank accession numbers (see Supplementary Files S1 and S2 online).

**Table 4:** Dentification and characterization of isolated strains based on 16S rRNA sequencing. The table includes GenBank accession numbers, microbiological characteristics, and the production activities of IAA-like compounds, both in the presence and absence of NaCl.

Strain* (accession number)	No. of the culture**	Cultural and morphological characteristics			IAA-like compounds production	
		Colony description	Colony shape and elevation	Gram reaction and motility	With NaCl (1 M)	Without NaCl
*Bacillus paramycoides* Lb-1 (PP087939)	1	Moderate size, viscid texture, creamy	Round, raised	Gram + , non-motile	–	+
*Phyllobacterium ifriqiyense* JS1 (PP087911)	4	Smaller size, viscid texture, whitish	Round, convex	Gram -, motile	–	+
*Pseudomonas koreensis* MPA1 (PP087927)	5	Smaller size, dry texture, translucent	Round, convex	Gram -, motile	+	+
*Arthrobacter subterraneus* Y1 (PP087959)	7	Smaller size, dry texture, yellowish	Round, convex	Gram + , non-motile	+	+
*Pseudomonas frederiksbergensis* AMA1 (PP087923)	9	Large size, smooth texture, translucent	Irregular, flat	Gram -, motile	+	+

* Identified percentages > 94% according to 16S rRNA sequencing.

** As shown in Fig. 1.

Genome atlases of the *Bacillus paramycoides* Lb-1 *and Phyllobacterium ifriqiyense JS1* are given as examples in the supplementary file section (see Supplementary File S3 online).

### Coal solubilization traits

**Fig. 3:**
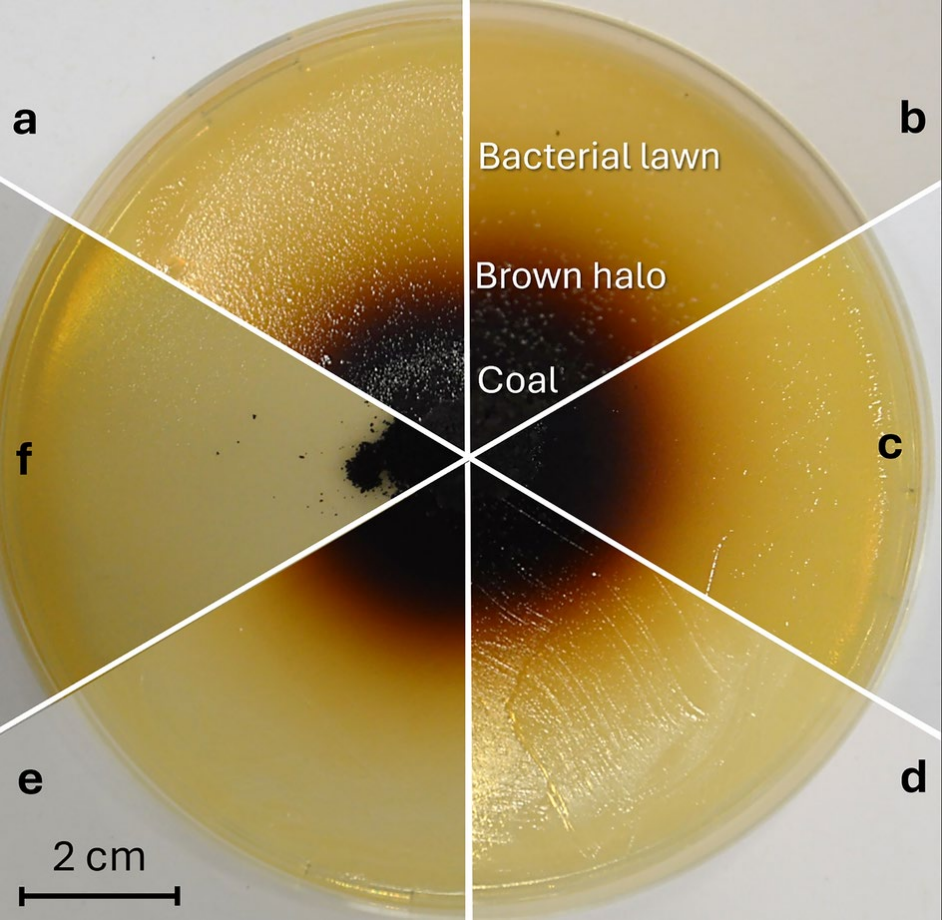
Coal biosolubilization by selected bacterial strains: (a) *Bacillus paramycoides* Lb-1, (b) *Phyllobacterium ifriqiyense* JS1, (c) *Pseudomonas koreensis* MPA1, (d) *Arthrobacter subterraneus* Y1, and (e) *Pseudomonas frederiksbergensis* AMA1. Panel (f) shows the control without bacterial inoculation.

Coal biosolubilization was first evaluated using a plate test (Fig. 3). All examined bacterial strains induced a noticeable change in the color of LB agar supplemented with coal after 7 days, indicating pronounced solubilization of the humic substances from the coal particles. The mean diameter of the brown halos reached approx. 45 mm. In contrast, no changes were detected in the non-inoculated control.

**Fig. 4:**
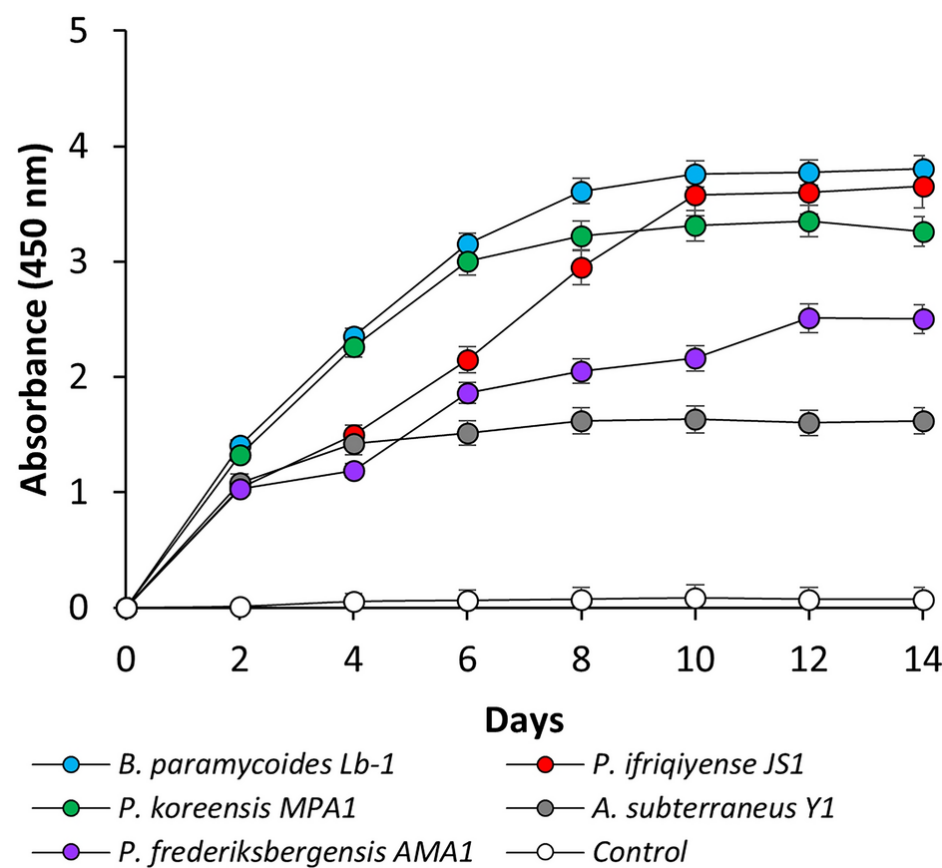
Degree of coal biosolubilization by different bacterial strains measured using the submerged cultivation method. Error bars represent the standard deviations for n = 3 replicates.

Next, the coal solubilization activity of the isolates was evaluated using a submerged cultivation method, determined by an increase in absorbance at 450 nm. The highest degree of biosolubilization occurred within the initial 10 days, potentially due to the inherently restricted dynamic range of the spectrophotometer. Coal solubilization activity was found in all five tested strains. The strain demonstrating the highest activity was *B. paramycoides* Lb-1, with absorbance values attaining 3.76 ± 0.3 during the 8^th^ day of cultivation (Fig. 4).

Both biosolubilization methods confirmed the metabolic capacity of the isolates to rapidly decompose the coal matrix. Minor changes in the absorbance values observed in the inoculum-free group (e.g. 0.08 ± 0.4 at day 10) may indicate that a minimal quantity of humic substances can be released from the lignite by the nutrient broth alone.

The observed temporal pattern of the A_450_ value suggests a correlation between biosolubilization activity and pH increase during cultivation (Table 5). The pH increase observed during biosolubilization may be the result of alkaline metabolites release by cultured cells. The initial pH of 6.83 increased to a maximum of 8.42 on day 10. In contrast, the inoculum-free culture medium (control) exhibited rather a modest pH reduction, likely due to the acidic properties of lignite^36^.

**Table 5:** Dynamics of culture media pH during coal biosolubilization by the bacterial isolates.

Days	*B. paramycoides* Lb-1	*P. ifriqiyense* JS1	*P. koreensis* MPA1	*A. subterraneus* Y1	*P. frederiksbergensis* AMA1	Control
0	6.83 ± 0.05	6.83 ± 0.01	6.83 ± 0.09	6.83 ± 0.04	6.83 ± 0.02	6.83 ± 0.03
2	7.48 ± 0.06	7.53 ± 0.01	7.58 ± 0.08	7.19 ± 0.05	7.56 ± 0.05	6.37 ± 0.00
4	7.96 ± 0.04	8.01 ± 0.07	8.20 ± 0.01	7.96 ± 0.02	8.17 ± 0.05	6.24 ± 0.08
6	8.16 ± 0.04	8.15 ± 0.03	8.17 ± 0.07	8.22 ± 0.08	8.11 ± 0.07	6.16 ± 0.02
8	8.38 ± 0.06	8.34 ± 0.08	8.33 ± 0.07	8.29 ± 0.01	8.26 ± 0.02	6.39 ± 0.04
10	8.42 ± 0.03	8.38 ± 0.08	8.33 ± 0.04	8.41 ± 0.07	8.15 ± 0.04	6.33 ± 0.02

**Fig. 5:**
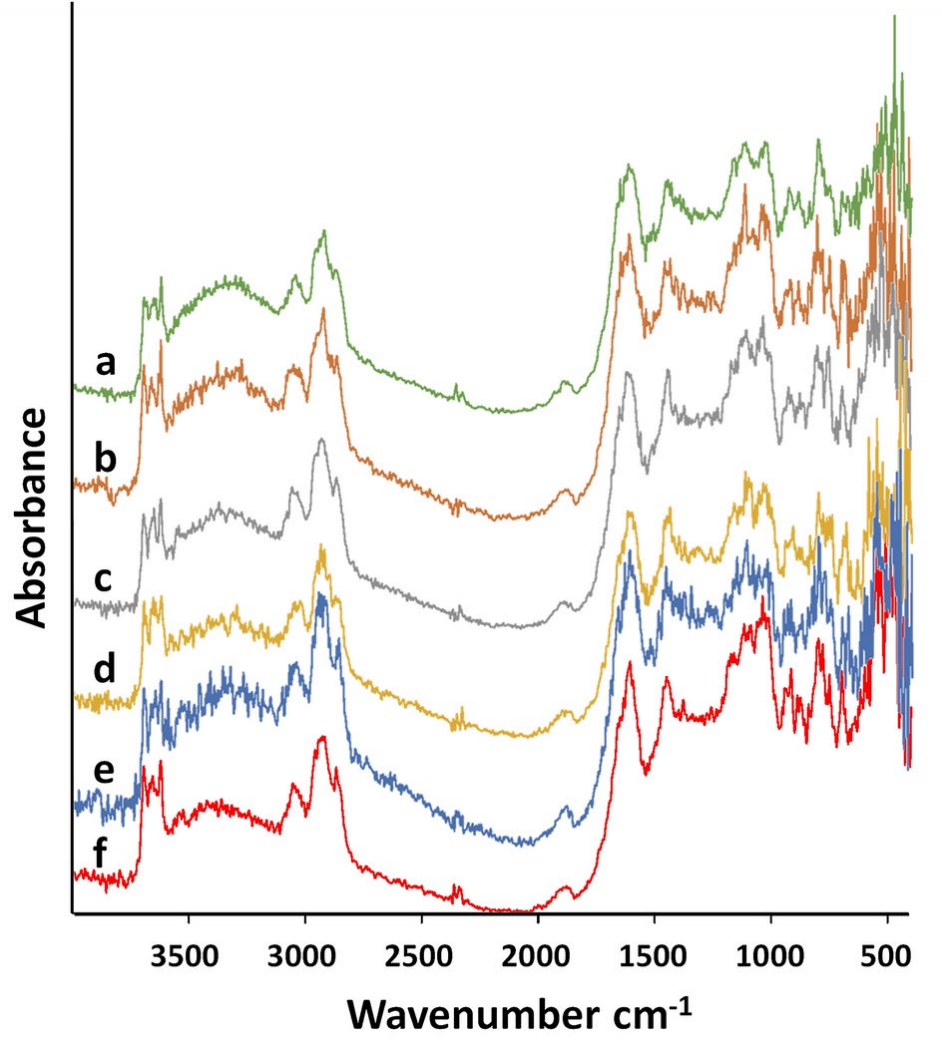
FTIR spectra of the coal biosolubilization products released after 10 days of incubation with the isolated strains. (**a**) *B. paramycoides* Lb-1, (**b**) *P. ifriqiyense* JS1, (**c**) *P. koreensis* MPA1, (**d**) *A. subterraneus* Y1, (**e**) *P. frederiksbergensis* AMA1, (**f**) control.

The FTIR spectra of the coal biosolubilization products revealed similar primary absorption bands with diverse functional groups (Fig. 5). The principal fingerprint regions correspond to -OH stretching vibrations (3700–1220 cm^-1^), alongside contributions from -NH_3_^+^, -NH_2_^+^, -CO–NH- and -CO-NH_2_^+^; aliphatic C-H bands (2980–2845 cm^-1^); carbonyl COOH, -CHO, and -CO- bands (1850–1660 cm^-1^), with minor contributions from esters. Further features include aromatic C = C and COO- bands (1635–1600 cm^-1^), less substituted rings (873–728 cm^-1^) and miscellaneous mineral compounds (430–550 cm^-1^). The IR spectrum of the control slightly differs from the biosolubilized coal spectra: for example, a band of moderate intensity at 1650 cm^-1^, prominent in the amide group (usually attributed to protein-like fragments), is absent in control. Furthermore, some low-intensity bands associated with oxygen-containing functional groups, such as carboxyl (1247 cm^-1^) and hydroxyl (1038 cm^-1^), are missing in the control sample, while bands at 3900, 3550, 1450 and 1150 cm^-1^ are, in contrast, more evident.

### Biomass production under saline conditions

**Fig. 6:**
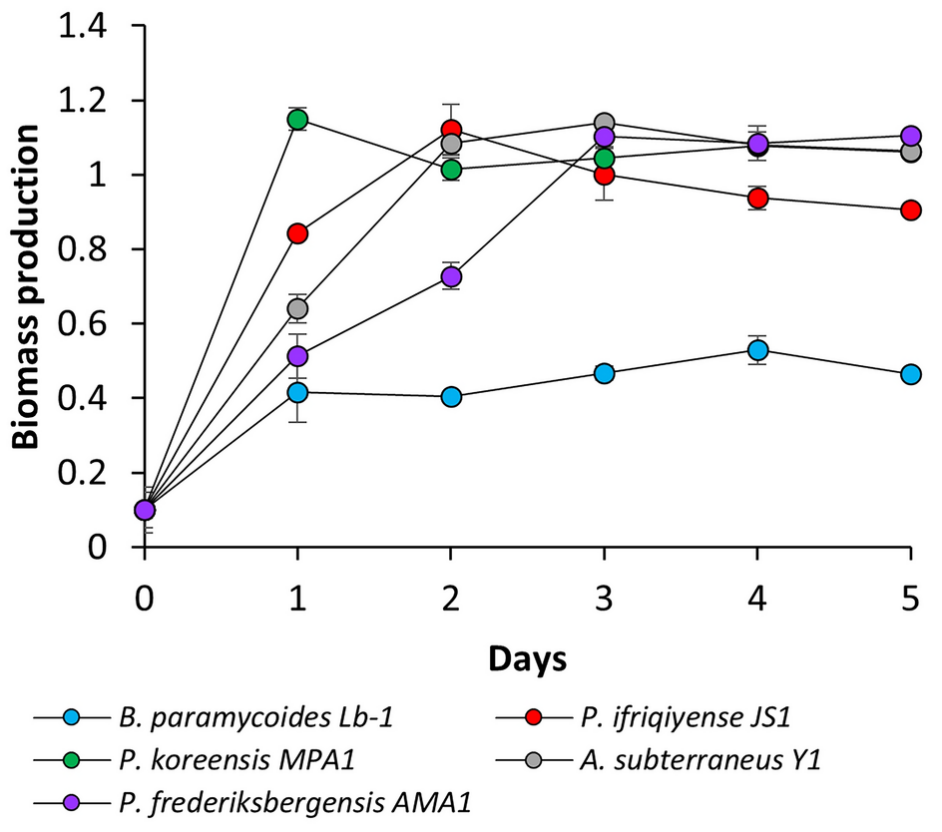
Dynamics of biomass production by strains isolated in media supplemented with 1 M NaCl.

All strains exhibited the ability to produce biomass under 1000 mM NaCl (Fig. 6). The strains *P. frederiksbergensis* AMA1, *P. koreensis* MPA1 and *A. subterraneus* Y1 exhibited the greatest biomass production and high salinity tolerance. Despite its low OD values, B. paramycoides Lb-1 consistently produced biomass at a stable rate in the saline medium.

### Effects of bacterial isolates on chia seedlings germination and growth

Incubation of the chia seeds with the isolated bacterial strains did not lead to significant differences (p > 0.05) in the proportion of seed germination between the different treatment types (Table 6). Both the test and control groups showed similar germination rates, exceeding 60%. However, a somewhat higher germination percentage resulting from *A. subterraneus* Y1 and *P. frederiksbergensis* AMA1 inoculations under all conditions should be noted.

**Table 6:** Germination percentages of chia seeds treated with bacterial strains under different conditions. The values were obtained from 10 seeds for each treatment variant.

Strains	Salt	Salt and coal	Coal
*B. paramycoides* Lb-1	60	80	60
*P. ifriqiyense* JS1	80	80	80
*P. koreensis* MPA1	80	60	60
*A. subterraneus* Y1	60	80	100
*P. frederiksbergensis* AMA1	80	60	80
Control	80	60	60

**Fig. 7:**
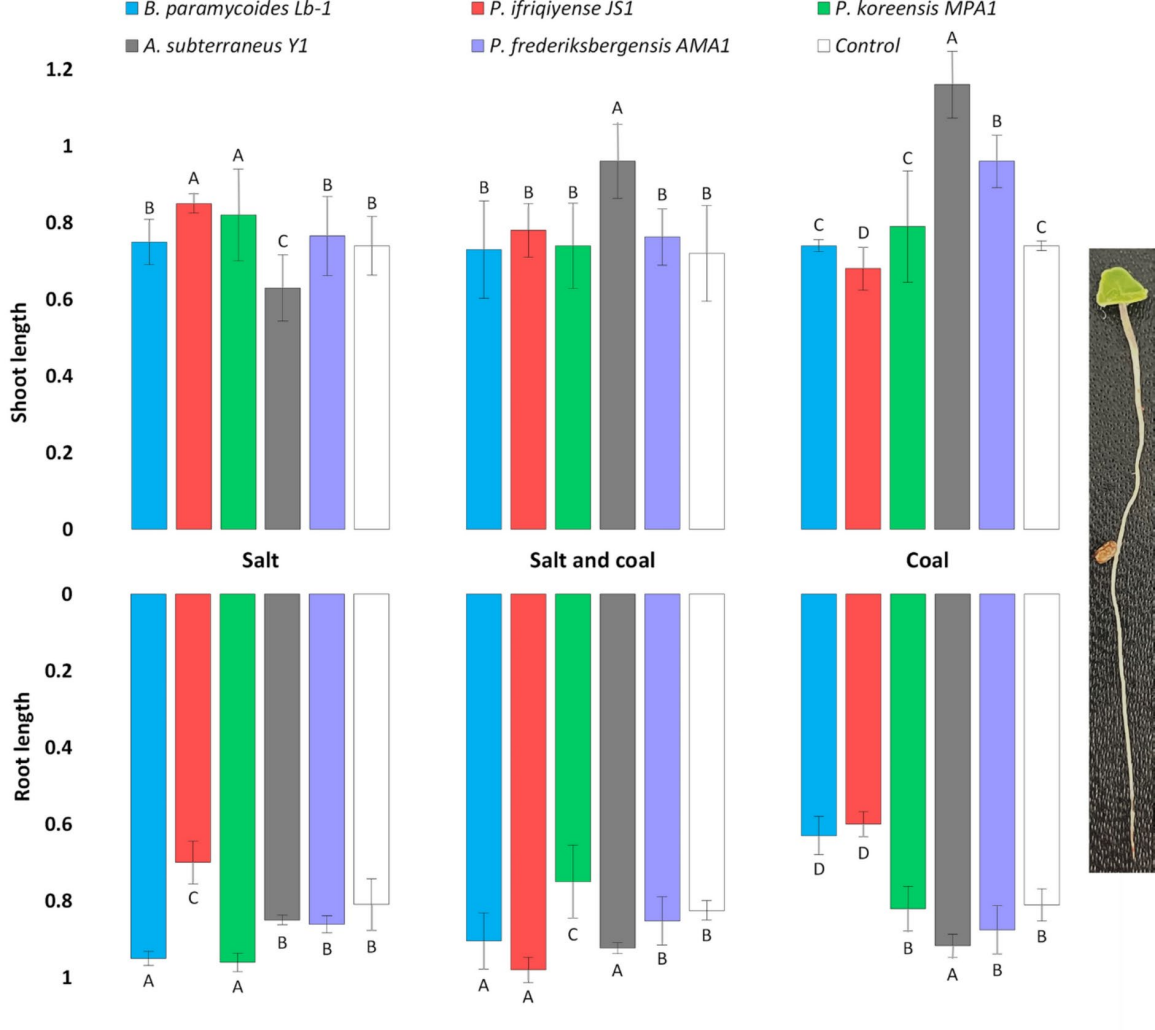
Root and shoot lengths of 7-day-old chia seedlings inoculated with isolated bacterial strains and grown on MS/2 agar under different salinity conditions. The error bars represent the standard deviation of the mean of the data. Capital letters denote the comparison of different inoculations under the same conditions (p ≤ 0.05).

Notably, greater differences in chia shoot length were observed between treatments (Fig. 7). Incubation with all strains, excluding *A. subterraneus Y1*, resulted in significantly increased initial chia growth under saline conditions (without coal) compared with the uninoculated control. Meanwhile, seedlings inoculated with *A. subterraneus* Y1 and *P. frederiksbergensis* AMA1 produced significantly longer shoots in both coal treatment groups. In *A. subterraneus Y1*, in particular, the adverse effects of high salinity seemed to be effectively mitigated by the bacterial inoculum.

The effect of salinity on root length was more variable and thus less conclusive. Roots were generally shorter when chia seedlings were inoculated with *B. paramycoides* Lb-1 and *P. ifriqiyense* JS1 under coal conditions, showing reductions of 22.2% and 25.9%, respectively, compared with the control. However, a (statistically significant) increase in root length was observed for both strains under coal-supplemented saline conditions.

### Mutual biocompatibility assessment

**Fig. 8:**
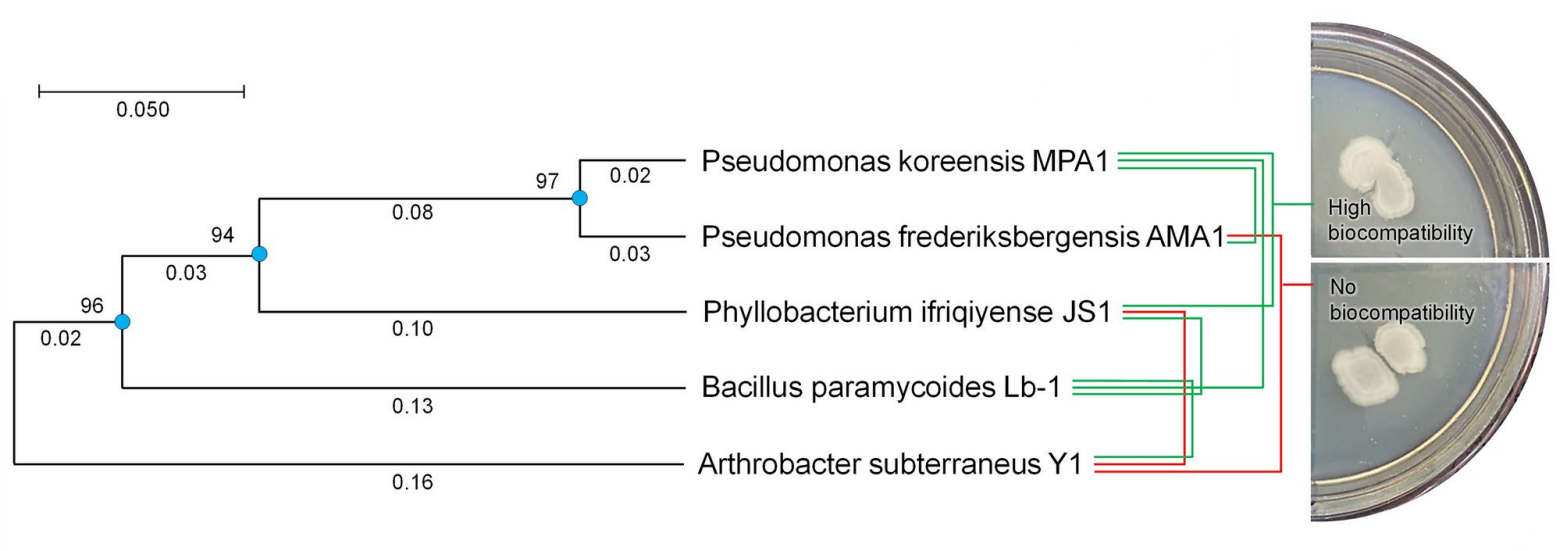
Biocompatibility study of the screened strains and their distant relationships (green line – high biocompatibility, red line—no biocompatibility).

The findings regarding the biocompatibility of the isolated strains are shown in Fig. 8.

Biocompatibility analysis revealed that most strains exhibited high biocompatibility, whereas some demonstrated antagonistic effects. In particular, the genetically most distant strain *A. subterraneus* Y1 exhibited poor biocompatibility with *P. ifriqiyense* JS1 and *P. frederiksbergensis* AMA1.

## Discussion

The soil used in this study was initially of low quality, as indicated by the results of bulk soil analysis. The soil samples exhibited typical characteristics of sandy soils, including low fertility (visible in situ), poor structure, and texture, primarily composed of sand with a small amount of clay. As predicted, the soil had an elevated EC value of 8.03 dS/m, corresponding to a moderate salinity level^37^.

We isolated soil microorganisms from the rhizospheres of *Artemisia annua* L. plants grown in saline soil and cultivated them in salt-containing coal nutrient media. The 14 isolated bacterial cultures from this soil type have been successfully adapted to coal substrates, which can serve as a source of organic matter and are shown to be beneficial for biological activity in the plant rhizosphere^11,38^. The phytoremediation potential of most *Artemisia* species in salt-impacted soils has been evaluated elsewhere^39^. This plant is a well-known facultative halophyte that can thrive in saline environments^40,41^.

Liu et al. argued that high-abundance bacterial communities are especially beneficial in saline-alkali soils by forming syntrophic relationships between *Artemisia* species and keystone taxa, which are crucial for establishing microbial network structures in the rhizosphere^42^. Previously, Alexander et al. reported that the rhizosphere of halophile plants facilitates the formation of microbial communities capable of enduring elevated salt concentrations, thereby benefiting the host plant and offering possible use in agriculture under saline stress^43^. The findings of Banerjee et al. suggested that *Pseudomonas* could benefit from such conditions, being a well-recognized and important PGP-active genus in the rhizosphere of saline environments^44^. Other PGP-relevant bacterial groups, including *Bacillus*, *Phyllobacterium*, and *Arthrobacter*, were also repeatedly isolated from saline soils, highlighting their ecological potential in various salt-affected soil scenarios^45–47^. The growth of salt-tolerant bacterial soil inhabitants on a coal medium represents yet another interesting ecological dimension, opening even more soil remediation options. Thus, such bacterial isolates are of great practical interest due to their plant growth-promoting and coal-solubilizing capabilities.

In the context of PGP-related screening, all 14 strains isolated in this study exhibited certain activity levels under saline conditions but only 5 of them possessed the full set of desired PGP activities. Nitrogen fixation and phosphate solubilization both with and without salt were particularly noteworthy among *Pseudomonas* species. Among all isolates, *Pseudomonas* produced siderophore and showed the highest biomass production under saline conditions. In line with our findings, presented here, various species of *Pseudomonas* were reported to possess a broad spectrum of PGP characteristics, including the production of IAA-like compounds, exopolysaccharide and siderophore in response to various stimuli in salt-supplemented media^48–50^. According to Amri et al., several *Pseudomonas* species exhibited the highest phosphate solubilization ability and greater pH reduction, indicating the high production of organic acids^51^. Similarly, in our study, *Pseudomonas koreensis* MPA1 exhibited the highest degree of PGP activities in the presence of 1 M NaCl. The capability of this strain to exhibit diverse PGP characteristics concurrently was also confirmed in two very recent studies^52,53^.

On the contrary, another isolate, *Bacillus paramycoides*, is a yet poorly studied representative of saline soil environments; however it showed great PGP potential. In our study, this strain exhibited nearly all PGP activities except the ability to produce IAA-like compounds under saline conditions. The preprint by Apaza-Castillo et al. presents the draft genome map of the *B. paramycoides* strain RZ3MS14, which displays several genes associated with PGP and resistance to abiotic stresses, thus highlighting the potential of this microorganism as a promising future tool in sustainable agriculture^54^. *Phyllobacterium ifriqiyense* JS1 also possessed most PGP characteristics except for IAA-like compounds synthesis under 1 M NaCl. As a member of rhizosphere microbial communities *P. ifriqiyense* is known to exhibit phosphate-solubilizing^55^, nitrogen fixation^56^, siderophore production^57^ and plant nodulation characteristics^58^. Finally, the *Arthrobacter subterraneus* Y1 isolate also demonstrated impressive PGP traits together with high biomass production under salinity conditions. Indeed, many *Arthrobacter* species were mentioned as halotolerant agents capable of alleviating plant stress to salinity, and genes associated with PGP activities in these species have been described by several authors^59–61^.

Regarding the PGP activity assessment and biomass production under saline conditions, we selected five best strains for further tests. The highest coal biosolubilization activity was observed for *B. paramycoides* Lb-1, whereas *A. subterraneus* Y1 showed a relatively low rate of coal decomposition. This result is not surprising, since *B. paramycoides* is indigenous to coal environments and was isolated from coal mining areas due to its bioremediation potential^62,63^. Moreover, the activity of *Bacillus* can be further enhanced to accelerate coal biotransformation, thereby aiding in the rehabilitation of saline-sodic soils^11^. Cubillos-Hinojosa et al. demonstrated that the addition of 1% low-rank coal and *Bacillus* sp. to saline-sodic soil resulted in enhanced soil respiration, cation exchange capacity, along with reductions in electrical conductivity, sodium adsorption ratio, and sodium saturation percentage^64^. However, prior to our study, there was no available literature data indicating that *A. subterraneus* alone can perform direct coal biosolubilization. In contrast, a plethora of studies have reported that *Pseudomonas* species can successfully metabolize low-rank coals^65,66^. The depolymerization of coal by Pseudomonas sp. involves a series of complex cleavage and modification processes targeting various chemical bonds, including ester and ether linkages, aromatic rings and internal carbon–carbon systems^67^. Consistent with our FTIR results, Malik et al. identified the major absorbance bands as corresponding to the carbonyl, hydroxyl, ether, carboxyl and side chains of the aromatic ring^68^.

The distinct correlation between pH value and the progress of in vitro bacterial coal biosolubilization, observed during our study, aligns with previously reported data^69,70^. It is widely assumed that coal solubilization typically results in the formation of dark-colored highly conjugated water-soluble HOM, with elevated pH values of 7–10 attributed to the presence of alkalic functional groups found in surfactants, chelating agents and some hydrolytic enzymes^71^.

González et al. highlighted the potential of *Pseudomonas* strains isolated from coal-mining areas to restore degraded soils and promote plant growth^72^. There is now considerable evidence that *Pseudomonas* and highly drought-tolerant *Phyllobacterium* species can play crucial roles in developing inoculant strategies aimed at rehabilitating saline-sodic soils and areas degraded by coal mining^55,73,74^. Inoculation of chia seeds with the isolated strains in our study showed different effects with respect to saline stress responses. The germination and growth parameters of seeds in all inoculation variants (except *A. subterraneus* Y1 groups) were higher than those of the control. The observed positive effects might be attributed to salt capture or exclusion mechanisms, which promote seed germination and plant growth, a phenomenon typically observed in microorganisms that produce extracellular polymeric substances^75^. Furthermore, the high biomass formation rate, observed in the majority of the strains, together with extracellular (secreted) biopolymers can also contribute to the mitigation of salinity stress by diminishing the amount of Na^+^ available for plant absorption, thereby promoting plant growth^76,77^.

Specifically, *P.ifriqiyense* JS1, a bacterium recognized for its abiotic stress tolerance and PGP characteristics, positively influenced the growth parameters and germination of chia in our experiments. However, the observed effect was reduced when the medium was amended with coal. As previously mentioned, this strain has not been extensively studied in coal utilization research. Yet in our tests, it was able to compensate for the biosolubilization effect, showing the second highest growth parameters in a salt-supplemented coal medium.

Interestingly, *A. subterraneus* Y1, a strain with a relatively low rate of coal biosolubilization, had the greatest positive effect on the germination and growth parameters of chia in coal and salt-supplemented coal media. Despite the lack of reports regarding the role of this strain in coal utilization, several studies indicated that *Arthrobacter* sp. possess distinct mechanisms of plant germination and growth biostimulation under certain nutrient availability^78,79^. The limited amount of solubilized coal products could be a favorable factor for *Arthrobacter* because these bacteria are able to utilize a wider range of organic substances as carbon and energy sources^80^. In addition, the efficacy of *Arthrobacter* species have been shown with respect of beneficial influence on root morphophysiological parameters under stress conditions^81^.

Positive reactions from chia seeds were also detected after inoculation with *P. frederiksbergensis* AMA1 in a salt-supplemented coal medium. The beneficial impact of this strain manifested in a more balanced proportion of shoot and root length compared with other inoculations. We believe this strain, with its great potential to enhance salinity tolerance and promote the growth of various plants, is a highly underrated bioinoculant, though there are initial signs of its scientific recognition^82^. Surprisingly, another *Pseudomonas* strain *P. koreensis* MPA1, which is closely related to *P. frederiksbergensis* AMA1, showed the opposite effect in a salt-supplemented coal medium, with lower seed growth parameters. In the case of *B. paramycoides* Lb-1, a moderate stimulating effect was observed for all treatment variants.

A consortium of mutually compatible rhizobacterial strains that promote plant growth has been shown to outperform the individual effects of the strains on plant growth parameters^83^. Indeed, it would be very attractive to combine the inoculants of interrelated traits in order to benefit from their collective advantages^84^. The PGP-exhibiting strains selected in our study may thus possess the potential to enhance plant germination and growth when combined synergistically with coal-solubilizing strains. Therefore, it was interesting to test the mutual compatibility of the isolated strains and assess their potential for creating ecological consortia. Our test revealed that *A. subterraneus* Y1, being a genetically distinct strain, exerted antagonistic effects against *P. ifriqiyense* JS1 and *P. frederiksbergensis* AMA1, as illustrated in a phylogenetic tree obtained via sequence analysis from the NCBI database. However, promising synergism was observed between the remaining bacterial strains demonstrating high degree of mutual biocompatibility.

Over the past two decades, the interaction between PGP bacteria and their host plants in coal-affected areas, as well as their responses to diverse factors, has garnered significant scientific interest^85,86^. Comprehending the mechanisms by which PGPB solubilize coal and enhance plant development provides critical insights for screening and managing plant species in the context of degraded land (re)vegetation and productivity. In general, coal solubilization refers to a nonenzymatic dissolution process that occurs at alkaline pH levels in the presence of alkaline substances, chelators, and surfactants, resulting in the formation of a dark liquid. Primarily, alkaline compounds, including ammonia, biogenic amines, peptides, and their derivatives, play significant roles in coal biosolubilization. These non-enzymatic compounds are synthesized by bacteria and fungi through the utilization of the organic acids present in the medium. They enhance the oxidation process by neutralizing the carboxylic acids found in coal, ultimately facilitating its solubilization^87–89^ Furthermore, biosurfactants synthesized by specific bacterial strains facilitate the solubilization and dissolution of coal by enhancing the adsorption of biological enzymes onto the coal surface and by diminishing surface tension. In addition, surfactants have the capacity to alter the reaction sites of specific enzymes, potentially enhancing the coal biodegradation rate^65,86,90^.

Comprehensive investigations concerning alkaline compounds and biosurfactants produced during coal biosolubilization should be undertaken to clarify the complex mechanisms involved in the solubilization of coal. The complete genome sequencing of both currently recognized and novel microorganisms engaged in biosolubilization could represent a significant advancement toward industrialization. However, no study examining the processes by which PGP bacteria directly solubilize lignite is available in open resources. Nonetheless, diverse PGPB frequently exhibit one or more mechanisms, resulting in certain bacteria being more adapted to specific environments, such as particular pH ranges, alongside a broad spectrum of PGPB, each varying in functionality under distinct environmental and soil conditions^91^. PGPB can stimulate plant growth through both direct and indirect mechanisms.

Direct mechanisms are characterized by the utilization of bacterial traits that enhance plant growth. Their functions encompass the synthesis of auxin, 1-aminocyclopropane-1-carboxylate deaminase, cytokinin, gibberellin, nitrogen fixation, phosphorous solubilization, and iron sequestration via bacterial siderophores. Indirect mechanisms denote bacterial characteristics that impede the activity of many plant pathogens, including both fungi and bacteria. Indirect mechanisms encompass antibiotics, cell wall-degrading enzymes, competition, hydrogen cyanide, induced systemic resistance, quorum quenching, and siderophores. Halotolerant PGP bacteria utilize a diverse array of strategies to adapt to saline environments, thereby facilitating various mechanisms that benefit plant growth in the context of salinity stress. Among these mechanisms is the modulation of the expression of stress-responsive genes, including DREB2b, RD29A, RD29B, and RAB18, which contribute to the enhancement of plant growth under adverse conditions^92–95^. In fact, no single bacterial species has the capability to utilize all pathways for lignite solubilization and plant growth stimulation^87^. Thus, microbial communities with high species diversity should be considered. To address the above-mentioned reasons, *our future research* is aimed at studying the combined effects of selected strains on coal solubilization and plant development and more accurately assessing their performance in natural settings.

## Conclusion

Novel strains of *Bacillus, Phyllobacterium, Arthrobacter,* and *Pseudomonas*, have been isolated and selected as efficient microorganisms exhibiting distinct PGP traits and significant coal biosolubilization under saline conditions. In the context of PGP screening, all isolates exhibited prominent activity for nitrogen fixation, phosphate solubilization, siderophore and IAA-like compound production, indicating their potential use in various scenarios of salt-affected soils. Their promising metabolic profiles were further validated in the context of coal biosolubilization, demonstrating additional functional properties for converting coal substrates into valuable humic products. To the best of our knowledge, this is the first study to report the screening and identification of strains with positive PGP and coal-solubilizing responses to saline stress. We expect that the isolated microbial strains may become a part of a more effective strategy for the reclamation of salt-affected soils and will help increase plant productivity.

## Supplementary Information

Supplementary Information 1.

Supplementary Information 2.

Supplementary Information 3.

## Data Availability

The datasets generated during and/or analysed during the current study are available from the corresponding author on reasonable request.
