# Supplementary material for: Screening halotolerant bacteria for their potential as plant growth-promoting and coal-solubilizing agents
Source: Sci Rep. 2025 Apr 16;15:13138. doi: 10.1038/s41598-025-98005-z (PMC12003788; doi:10.1038/s41598-025-98005-z)
Supplement: Supplementary file 1 — Supplementary Information 1. [file 41598_2025_98005_MOESM1_ESM.docx]

**Supplementary File S1**

Identification results of the isolated strains based on 16S rRNA sequencing (including GenBank accession numbers)

| **Strain** | **Accession number** | **Links from NCBI** |
| --- | --- | --- |
| *Bacillus paramycoides* strain Lb-1 | PP087939 | <https://www.ncbi.nlm.nih.gov/nuccore/PP087939.1/> |
| *Phyllobacterium ifriqiyense* strain JS1 | PP087911 | <https://www.ncbi.nlm.nih.gov/nuccore/PP087911.1/> |
| *Pseudomonas koreensis* strain MPA1 | PP087927 | <https://www.ncbi.nlm.nih.gov/nuccore/PP087927.1/> |
| *Arthrobacter subterraneus* strain Y1 | PP087959 | <https://www.ncbi.nlm.nih.gov/nuccore/PP087959.1/> |
| *Pseudomonas frederiksbergensis* strain AMA1 | PP087923 | <https://www.ncbi.nlm.nih.gov/nuccore/PP087923.1/> |
