# Supplementary material for: Screening halotolerant bacteria for their potential as plant growth-promoting and coal-solubilizing agents
Source: Sci Rep. 2025 Apr 16;15:13138. doi: 10.1038/s41598-025-98005-z (PMC12003788; doi:10.1038/s41598-025-98005-z)

**Supplementary File S2**

The phylogenetic tree of the strains were constructed using the Neighbor-Joining (NJ) method using MEGA v.11 by MEGA Software Development Team (https://www.megasoftware.net).

***Bacillus paramycoides strain Lb-1***

*
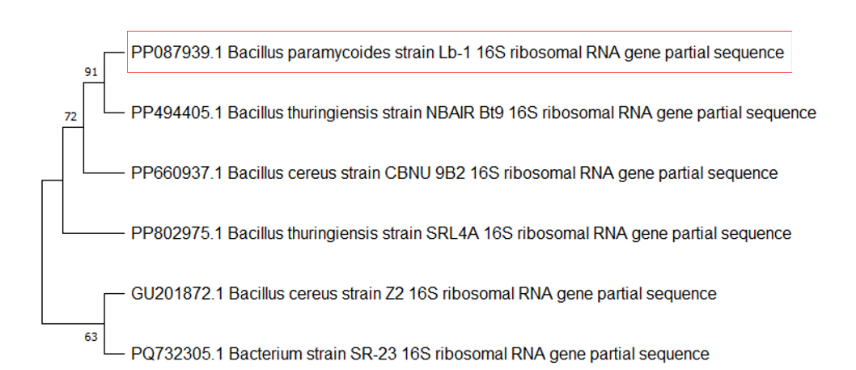
*

***Phyllobacterium ifriqiyense strain JS1***


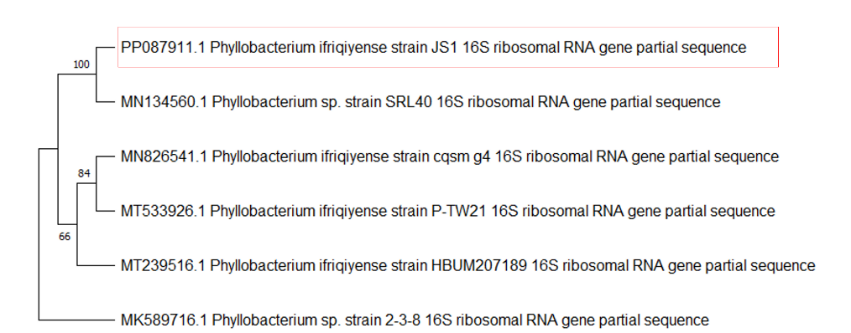


***Pseudomonas koreensis strain MPA1***

*
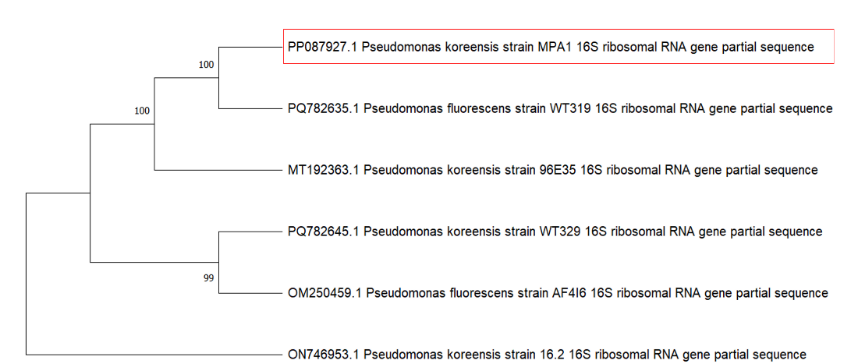
*

***Arthrobacter subterraneus strain Y1***


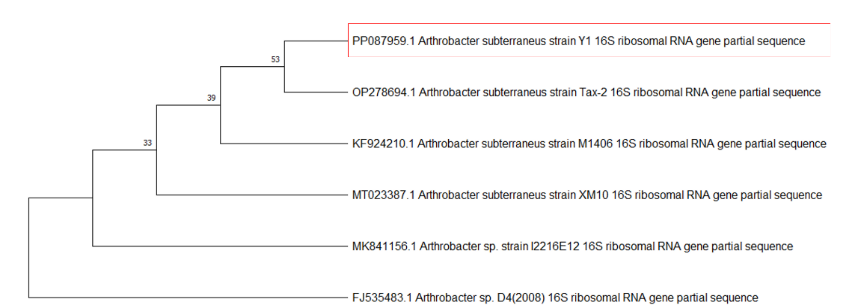


***Pseudomonas frederiksbergensis strain AMA1***


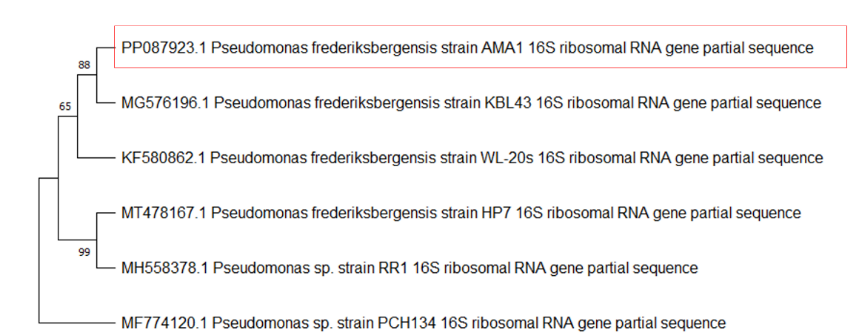

Supplement: Supplementary file 2 — Supplementary Information 2. [file 41598_2025_98005_MOESM2_ESM.docx]
