# Supplementary material for: Screening halotolerant bacteria for their potential as plant growth-promoting and coal-solubilizing agents
Source: Sci Rep. 2025 Apr 16;15:13138. doi: 10.1038/s41598-025-98005-z (PMC12003788; doi:10.1038/s41598-025-98005-z)

**Supplementary File S3**

Genome atlases of the *Bacillus paramycoides* Lb-1 and *Phyllobacterium ifriqiyense* JS1. The diagrams were created with Circos Software v.0.69-9 created by Martin Krzywinski (https://circos.ca). A comprehensive interpretation of the genomic data is planned for a subsequent publication.


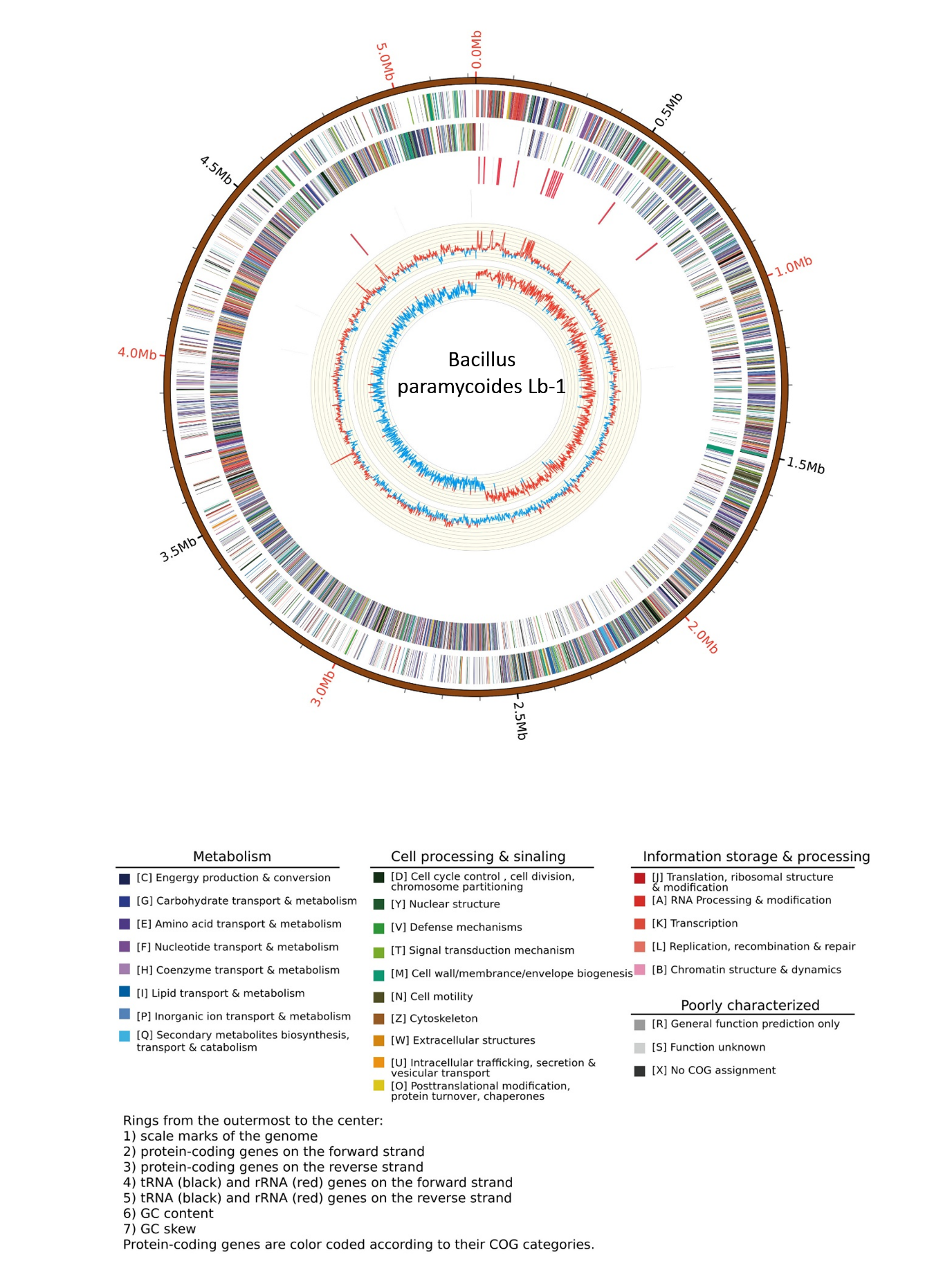


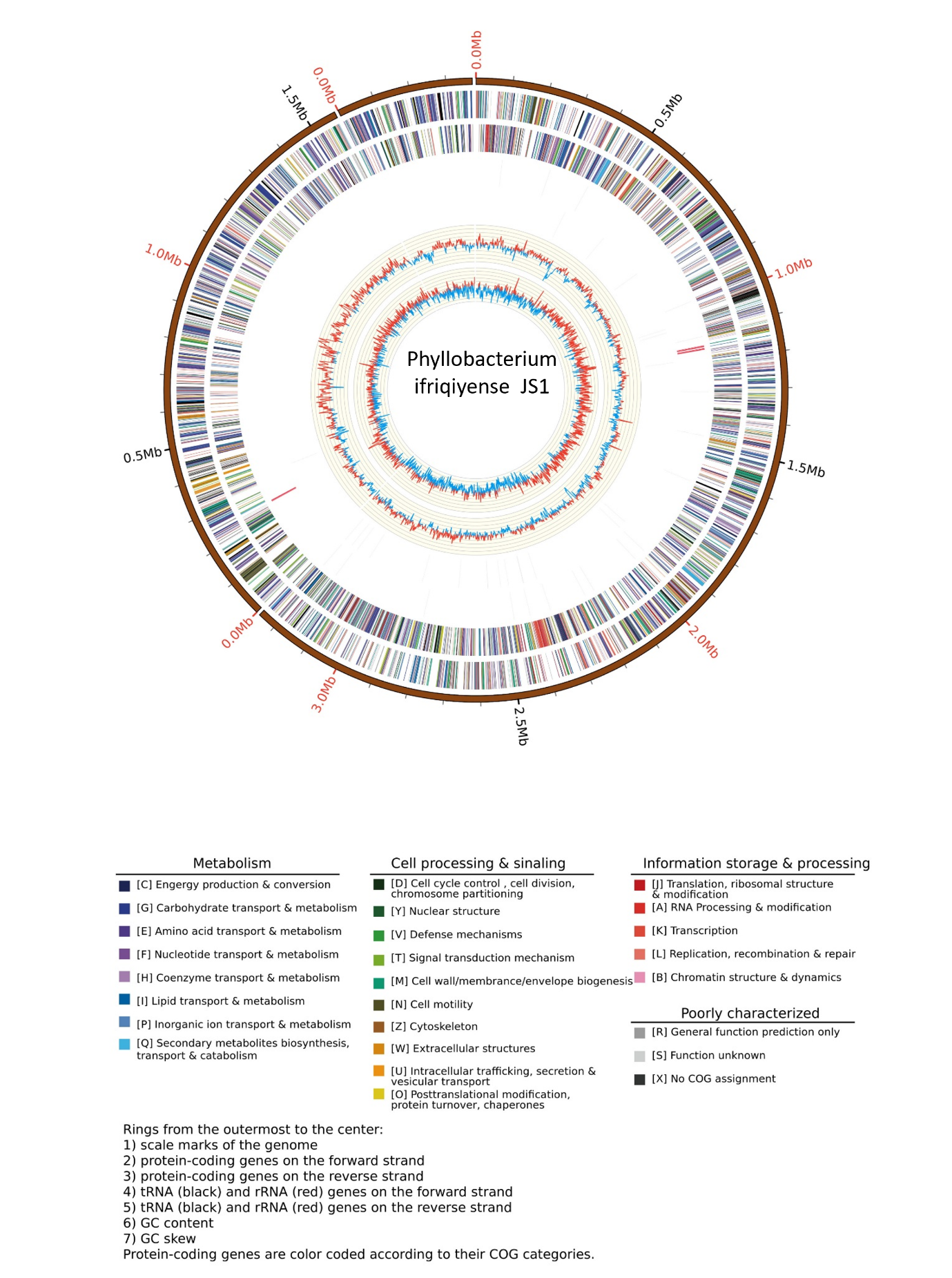

Supplement: Supplementary file 3 — Supplementary Information 3. [file 41598_2025_98005_MOESM3_ESM.docx]
